# Genome-Wide Identification of MATE Gene Family in Potato (*Solanum tuberosum* L.) and Expression Analysis in Heavy Metal Stress

**DOI:** 10.3389/fgene.2021.650500

**Published:** 2021-05-28

**Authors:** Yun Huang, Guandi He, Weijun Tian, Dandan Li, Lulu Meng, Danxia Wu, Tengbing He

**Affiliations:** ^1^College of Agricultural, Guizhou University, Guiyang, China; ^2^Institute of Agro-Bioengineering, Guizhou University, Guiyang, China; ^3^Key Laboratory of Plant Resource Conservation and Germplasm Innovation in Mountainous Region, Ministry of Education, Guizhou University, Guiyang, China; ^4^College of Life Sciences, Guizhou University, Guiyang, China; ^5^Institute of New Rural Development, Guizhou University, Guiyang, China

**Keywords:** potato, heavy metals, MATE genes, phylogenetic relationship, expression analysis

## Abstract

A genome-wide identification and expression analysis of multidrug and toxic compound extrusion (MATE) gene family in potato was carried out to explore the response of MATE proteins to heavy meta stress. In this study, we identified 64 MATE genes from potato genome, which are located on 12 chromosomes, and are divided into I–IV subfamilies based on phylogenetic analysis. According to their order of appearance on the chromosomes, they were named from *StMATE1–64*. Subcellular location prediction showed that 98% of them are located on the plasma membrane as transporters. Synteny analysis showed that five pairs of collinearity gene pairs belonged to members of subfamily I and subfamily II had two pairs indicating that the duplication is of great significance to the evolution of genes in subfamilies I and II. Gene exon–intron structures and motif composition are more similar in the same subfamily. Every StMATE gene contained at least one *cis*-acting element associated with regulation of hormone transport. The relative expression levels of eight StMATE genes were significantly upregulated under Cu^2+^ stress compared with the non-stress condition (0 h). After Cd^2+^ stress for 24 h, the expression levels of *StMATE33* in leaf tissue were significantly increased, indicating its crucial role in the process of Cd^2+^ stress. Additionally, *StMATE18*/*60*/*40*/*33*/*5* were significantly induced by Cu^2+^ stress, while *StMATE59* (II) was significantly induced by Ni^2+^ stress. Our study initially explores the biological functions of StMATE genes in the regulation of heavy metal stress, further providing a theoretical basis for studying the subsequent molecular mechanisms in detail.

## Introduction

Various types of abiotic stresses, especially heavy metal pollution, which is also the main environmental problem, restrict plant growth (Mustafa and Komatsu, 2016). Unfortunately, plants absorb heavy metals along with essential elements from the soil; this caused them to evolve different strategies to deal with the detrimental accumulation of heavy metals. For example, heavy metal ATPase (HMA), multidrug and toxic compound extrusion (MATE), yellow stripe-like (YSL), and metal tolerance proteins (MTP) families are constitutively overexpressed transmembrane transport proteins that drive the uptake of heavy metals, transport them to the leaves to finally be sequestered in the vacuoles or cell walls (Rascio and Navari-Izzo, 2011). MATE transporters are ubiquitously distributed in plants (Omote et al., 2006) and are involved in a diverse array of functions encompassing secondary metabolite transport, xenobiotic detoxification, disease resistance, and aluminum tolerance (Upadhyay et al., 2019). In 1998, the first MATE protein, Norm, was cloned from the chromosomal DNA of *Vibrio parahaemolyticus* (Morita et al., 1998). Consequent experiments suggested that Norm gene belongs to the efflux protein gene and has a multidrug efflux function. Notably, most characterized MATE transporters seem to only export compounds with positive charges (Lu, 2016).

The members of the MATE family are reported to be directly or indirectly involved in the transit processes of disease resistance, aluminum detoxification, toxic metal efflux, secondary metabolites, and plant hormones. As a result, specific functions of some MATE genes in *Arabidopsis* have already been characterized. For instance, *AtDTX1* not only participates in the efflux of alkaloids, antibiotics, and other toxic compounds but also detoxifies Cd^2+^, a heavy metal (Li et al., 2002). *AtALF5* (*AtDTX19*) is reported to have similar functions as *AtDTX1* (Diener and Fink, 2001). It is very well known that *ADS1* can regulate plant disease resistance by encoding the MATE transporter (Sun et al., 2011). Interestingly, MATE protein also regulates the overall development of plants by controlling the phytohormone transfer. For example, *AtDTX50* can efflux ABA (Zhang et al., 2014), and both *AtFRD3* (*AtDTX43*) (Durrett et al., 2007) and *OsERDL1* (Yokosho et al., 2009) show citric acid activity and can transport metallic iron. Moreover, salicylic acid depends on *EDS5* to transmit the signals for disease resistance (Nawrath et al., 2002). TT12 (*AtDTX41*), the first MATE protein known to transport flavonoids, can also mediate the transport of anthocyanin cyanidin-3-O-glucoside in the presence of Mg-ATP (Marinova et al., 2007). On the other hand, *AtDTX18* can enhance plant defense against pathogens by transporting hydroxycinnamic acid amide (Dobritzsch et al., 2016). The function of MATE proteins in other plants has also been characterized, and it is known that *SbMATE* (Sivaguru et al., 2013), *ZmMATE1* and *ZmMATE2* (Maron et al., 2010), and *OsFRDL4* (Yokosho et al., 2011) all participate in the detoxification process of aluminum. Additionally, *OsMATE2* regulates the accumulation of arsenic in rice and tobacco (Das et al., 2018). Both *NtMATE1* and *NtMATE2* transport alkaloids to the vacuole, thus reducing their toxicity (Shoji et al., 2009). Alfalfa *MtMATE67* can enhance symbiotic nitrogen fixation by mediating citrate transport into the symbiotic plastid (Kryvoruchko et al., 2018). In addition, a study has shown that the overexpression of cotton MATE gene can regulate the amount of reactive oxygen species (ROS), thus minimizing the effects of various oxidative stresses (Lu et al., 2019).

Multidrug and toxic compound extrusion protein has been widely studied in several plants, such as rice (*Oryza sativa*) (Tiwari et al., 2014), *Arabidopsis* (*Arabidopsis thaliana*) (Wang et al., 2016), soybean (Liu et al., 2016), flax (Lu, 2016), upland cotton (Xu et al., 2019), tomato (Santos et al., 2017), maize (Zhu et al., 2016), alfalfa (Min et al., 2019), *Vitis vinifera* (Gomez et al., 2009), blueberry (Chen et al., 2015), etc. The functions of some of the MATE genes have been characterized, and all of these indicate that the MATE gene family plays an important role in plant growth, development, and stress resistance. The potato, an edible tuber, is the fourth most important food crop in the world. Many gene families have been identified and analyzed in potato, such as MYB gene family (Sun et al., 2019), StGRAS (Wang et al., 2019), heat shock proteins20 (Hsp20) (Zhao et al., 2018), HMA (He et al., 2020), ATP-binding cassette (ABC) (He et al., 2021), and so on. Recently, Li et al. (2019) and Chen Q. et al. (2020) conducted a preliminary identification of the MATE gene family in potatoes. However, the research on the response of MATE protein to heavy metals in potato is rare.

Soil contamination by heavy metals is a growing problem to human and animal health. To unearth the MATE gene in potato that responds to heavy metal stress, we set out to identify and analyze the MATE gene family in a potato type, *Cloud S. tuberosum 505*. Our study focused on MATE gene structure, chromosomal localization, phylogenetic relationship, analysis of *cis*-acting element, and expression level analysis following exposure to heavy metal stress.

## Materials and Methods

### Plant Materials and Treatments

*Cloud S. tuberosum 505* was selected as the test species, and potted planting was carried out in the Institute of New Rural Development of Guizhou University. At 21 days of seedling growth, potato plants with the same growth were selected and exposed to five heavy metals, namely, Cd^2+^(CdCl_2_), Cu^2+^(C_4_H_6_CuO_4_⋅H_2_O), Pb^2+^[Pb(CH_3_COO)_2_⋅3H_2_O], Ni^2+^[Ni(NO_3_)_2_⋅6H_2_O], and Zn^2+^(ZnCl_2_) at a concentration of 100 mg/kg as in a previous study (Tian et al., 2021). All of them are analytical reagents. Prepare five heavy metal solutions (Cd^2+^, Cu^2+^, Pb^2+^, Ni^2+^, and Zn^2+^) with a concentration of 100 mg/kg, and then soaking the soil with liquid solutions of metals. The roots, stems, and leaves of the seedlings were collected when treatment for 6, 12, and 24 h, place them into the foam box containing liquid nitrogen straightaway, finally laying in −80°C ultralow-temperature refrigerator.

### Identification and Analysis of MATE Gene Family

Downloading the genome annotation and protein files of *O. sativa*, *S. tuberosum*, and *A. thaliana*^1^ and the matrix file of hidden Markov model (HMM) of MATE gene family^2^. Then, we retrieve MATE gene family information from potato protein sequence database by the HMM search program of HMMER (v3.1) in a Linux system. Here, 1.2e-28 is set as “*E*-value” to obtain a reliable MATE domain, and multiple sequence alignment was carried out by ClustalW (v2.1). On the basis of the multiple sequence alignment, the specific MATE domain HMM of potato was constructed using the HMM build program of HMMER. HMM search was then again performed to retrieve the sequences with *E*-value (<0.001) in the protein file to obtain candidate MATE gene sequences. SMART^3^, CDD^4^, and PFAM^5^ were used to test the candidate protein sequences of potato MATE genes, and verification of the MATE domain was performed. Here, 64 MATE genes in total were identified in potato. The molecular weight, theoretical pI, number of amino acids, instability index (II), and grand average of hydropathicity (GRAVY) were all estimated with ExPASy^6^. The subcellular location prediction of StMATE proteins was determined with Plant-mPLoc^7^.

### Chromosome Distribution and Synteny Analysis

We obtained the position information of the MATE genes of potato on the chromosome by Linux system and then mapped the MATE genes on their chromosomal location by using MapChart^8^. The genes were named *StMATE1–64* according to the sequence of their appearance on the chromosome. In addition, we used MCScanX (Wang et al., 2012) to construct the gene synteny landscape.

### Phylogenetic Analysis

The MATE proteins identified in *S. tuberosum* (64), *A. thaliana* (56) (Wang et al., 2016), and *O. sativa* (45) (Li et al., 2002) were all aligned using the MEGA X (v10.0.2) with their default parameters. The tree of phylogenetic relationship was inferred by the NJ (neighbor-joining) method of MEGA X (v10.0.2), while the value of bootstraps was set to 1,000 to evaluate the reliability of internal branches. Additionally, Evolview^9^ was used to visualize the phylogenetic tree.

### Transmembrane Helices, Gene Structure, and Motif Analysis

Using MEME (v4.12.0) to extract MATE domains and search for 20 conservative motifs. We used TBtools (v1.0692), as previously mentioned (Chen C. et al., 2020) was for gene structure analysis and motif visualization. Protter^10^ is used to predict protein topology.

### Promoter Analysis

Promoter sequence analysis was performed in Plantcare^11^. Briefly, the 2,000-bp sequence upstream of the ATG start codon of the MATE gene family was taken as promoter sequence. *Cis*-acting elements that are not related to heavy metal stress were removed, and TBtools was used to draw and visualize the elements.

### Quantitative Real-Time PCR Analysis

To analyze the expression patterns of MATE genes of potato under different heavy metal stresses, we selected eight StMATE genes for Cd^2+^, Cu^2+^, Pb^2+^, Ni^2+^, and Zn^2+^ stress treatment. The selection of eight genes is based on collinearity analysis of MATE genes in rice, potato, and *Arabidopsis* (Table 1), preliminarily determined *StMATE33*-PGSC0003DMT400078371 (collinearity with *Arabidopsis* and rice MATE genes). Then, according to the *cis*-acting elements (*cis*-acting regulatory element involved in the MeJA-responsiveness and *cis*-acting element involved in defense and stress responsiveness) contained in *StMATE33*, combined with *cis*-acting elements (Figure 6) and phylogenetic tree (Figure 4) analysis, the eight selected genes were finally determined for further research. We analyzed the relative expression levels of StMATE genes in different tissues (root, stem, and leaf) by quantitative real-time PCR (qRT-PCR). The eight selected StMATE genes are, respectively, *StMATE33*/*40* (I), *StMATE45*/*59* (II), *StMATE5*/*61* (III), and *StMATE18*/*60* (IV). Primer Premier 6 was used to design the qRT-PCR primers for the eight MATE genes (Table 2). Primers were synthesized by Sangon Biotech (Shanghai, China) Co., Ltd. A TRIzol kit was used to extract total RNA and convert it to cDNA according to the manufacturer’s instruction (StarScript II First-Strand cDNA Synthesis Mix) after the removal of gDNA. The synthesized cDNA was stored in the refrigerator (−80°C) until further use. Here, 2 × RealStar Green Fast Mixture was used to perform qRT-PCR in accordance with the manufacturer’s instructions, and actin was used as the reference gene. The total volume of qRT-PCR reaction (20 μl), included 10 μl of 2 × RealStar Green Fast Mixture, 2 μl of cDNA template, 7 μl of ddH_2_O, and 1 μl of primers (0.5 μl each for forward and reverse primer) to a final concentration of 10 μM. The PCR reaction was carried out on a BIO-RAD CFX96 (Bio-Rad, United States) real-time fluorescent quantitative PCR instrument by a two-step method with the reaction parameters set as follows: 95°C for 3 min, 40 cycles of 95°C for 15 s, 60°C for 20 s. Three technical and three biological replicates were analyzed for each sample, and the analysis for qRT-PCR was calculated by the Ct comparison (2^–ΔΔ*Ct*^) method (Livak and Schmittgen, 2001). Cytoscape (v3.6.1) (Shannon et al., 2003) is used to establish and analyze the co-expression network of the eight StMATE genes.

**TABLE 1:** Twenty-five collinear gene pairs of multidrug and toxic compound extrusion (MATE) genes in *Arabidopsis*, potato, and rice.

**Gene pairs**	**Gene ID of potato**	**Gene ID**	**Gene pairs**	**Gene ID of potato**	**Gene ID**
1	PGSC0003DMT400005437	AT5G65380	14	PGSC0003DMT400026353	AT4G25640
2	PGSC0003DMT400022543	AT3G21690	15	PGSC0003DMT400072250	AT3G03620
3	PGSC0003DMT400078371	AT1G71140	16	PGSC0003DMT400014431	AT1G73700
4	PGSC0003DMT400010077	AT4G22790	17	PGSC0003DMT400017707	AT1G51340
5	PGSC0003DMT400078714	AT1G66780	18	PGSC0003DMT400042439	AT1G61890
6	PGSC0003DMT400058542	AT4G23030	19	PGSC0003DMT400005437	Os08t0480000
7	PGSC0003DMT400043015	AT1G66780	20	PGSC0003DMT400078371	Os01t0684900
8	PGSC0003DMT400041642	AT5G10420	21	PGSC0003DMT400078714	Os01t0684900
9	PGSC0003DMT400029423	AT5G49130	22	PGSC0003DMT400037015	Os10t0523201
10	PGSC0003DMT400047906	AT1G12950	23	PGSC0003DMT400041642	Os08t0480000
11	PGSC0003DMT400015300	AT1G71140	24	PGSC0003DMT400026353	Os02t0821600
12	PGSC0003DMT400032971	AT1G11670	25	PGSC0003DMT400005431	Os03t0188100
13	PGSC0003DMT400065544	AT3G21690			

**TABLE 2:** Primer design of eight genes.

**Primer name**	**Reverse primer (5′–3′)**	**Forward primer (5′–3′)**
Actin	AGCCACCACTGAGCACAATGTTAC	AGAGGTTCCGTTGCCCAGAGG
StMATE40	GCAGTACGCGCTACAAGACGAG	TGTTGCCTCAAGGACTAGGTGGAG
StMATE33	GCGATGGGAATGCAAGCAATGAC	ACTCTTTGCGGTCAGGCTTTCG
StMATE59	AGTGACCAACATCGTCCCATTTCC	GGCTGGAATGATCTTCGGTGGAAC
StMATE45	GCCAAACGTCCCCTTAGGAAGAAG	GAGTGCAGCATGTGTACGAGTAGC
StMATE5	CTTCTGCTGACATCGGTGGAATCC	CTGCGTACATTTGCCGGTTTATGG
StMATE61	TGGCTTTCGTTGTCTCGTGGTTC	GCTGCTGTGGGAATTTCGATTGC
StMATE18	CCAAACCCCACTCATAGCAACTCC	ACTCTGCCTCTGACTTGTTGTGC
StMATE60	TCCAATCCCATCCGTCCATCTCC	AGTTGCTATGGCTTCAGTGCTGAC

## Results

### Identification and Related Information of MATE Gene Family in Potato

A total of 64 MATE genes were identified by homology search and MATE domain analysis from potato genome, which were named *StMATE1–64* based on their location on the chromosome. MATE proteins of potato encoded by 64 StMATE genes were sequence analyzed, and the proteins were found to be 186–551 aa long, with the maximum molecular weight as 60.28 KD, and their isoelectric points ranged from 5.29 to 9.44 (Supplementary Table 1). The results of subcellular localization prediction showed that MATE proteins of potato are well distributed in plasma membrane, chloroplast, vacuole, and mitochondria. Interestingly, 98% of them are located on the plasma membrane, and 9.4% are distributed in both chloroplast and plasma membrane. However, *StMATE38* is specifically distributed across plasma membrane, vacuoles, and mitochondria.

### Chromosome Distribution, Collinearity, and Synteny Analysis of MATE Genes in Potato

**FIGURE 1:**
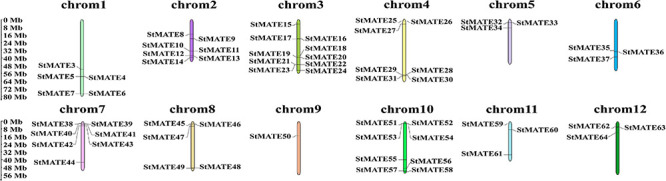
Chromosomal distribution of StMATE genes. The scale bar on the left represents the length (Mb) of the potato chromosomes and multidrug and toxic compound extrusion (MATE) genes are marked on both the sides of the respective chromosome.

**FIGURE 2:**
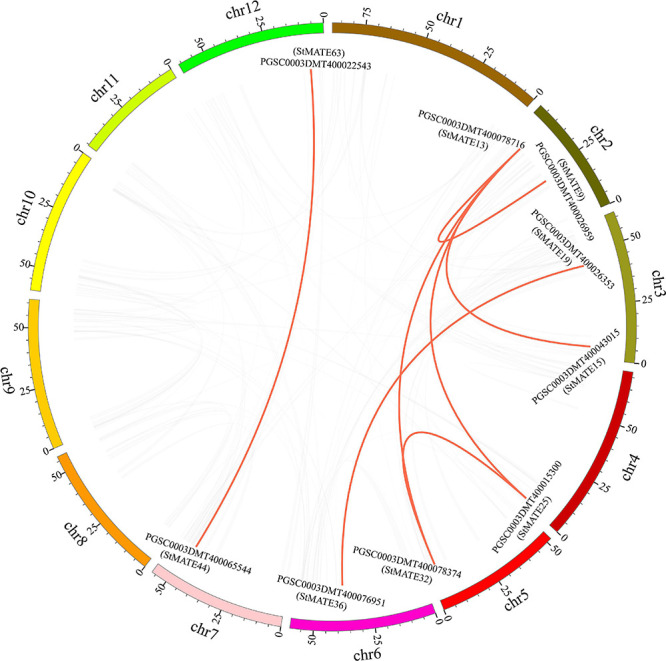
Syntenic relationships of StMATE genes. Potato chromosomes are shown in different colors. The putative orthologous multidrug and toxic compound extrusion (MATE) genes of potato are represented in red. The gene names are indicated inside of the diagram, while the chromosome numbers are shown outside the diagram.

**FIGURE 3:**
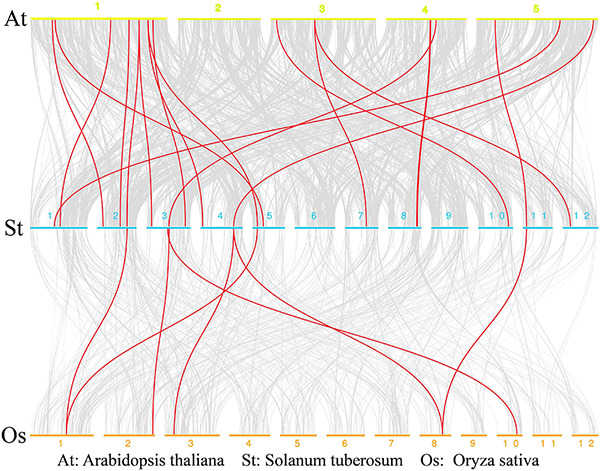
Collinearity analysis of multidrug and toxic compound extrusion (MATE) genes in rice, potato, and *Arabidopsis*. Chromosomes of *Arabidopsis*, potato, and rice are represented by yellow, blue, and orange bars, respectively; the chromosome label is next to the corresponding chromosome. The red curve indicates MATE genes with collinearity.

The 64 StMATE genes were distributed along the lengths of 12 chromosomes (Figure 1). We found 10 StMATE genes on chromosome 3 but only one (*StMATE50*) on chromosome 9. The result of synteny analysis of 64 StMATE genes demonstrated seven pairs of duplicated genes (Figure 2), namely, *StMATE44/63*, *StMATE19/36*, *StMATE25/32*, *StMATE13/15*, *StMATE13/25*, *StMATE13/32*, and *StMATE13/9*. Additionally, collinearity analysis showed that there are 18 pairs of collinearity genes of MATE gene family between potato and *Arabidopsis* but only seven pairs in potato and rice (Figure 3), gene IDs of 25 pairs of genes are shown in Table 1. It indicated that the collinearity between potato and *Arabidopsis* is more significant than that between potato and rice.

### Phylogenic Analysis and Classification

**FIGURE 4:**
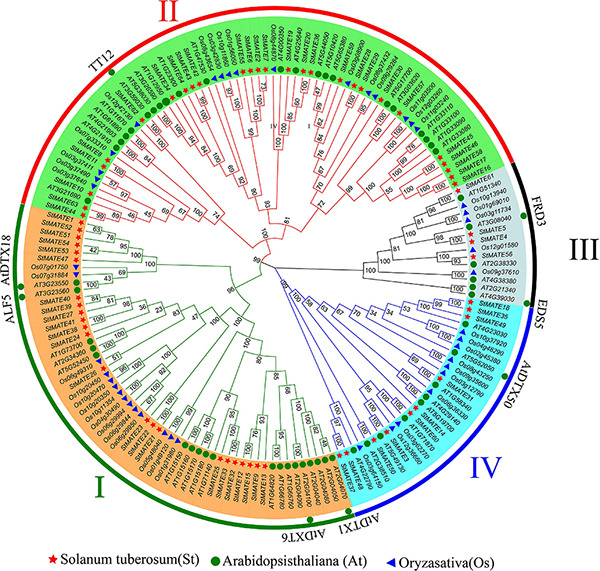
Phylogenetic tree of *Arabidopsis*, rice, and potato multidrug and toxic compound extrusion (MATE) proteins. The picture represents the phylogenetic tree as constructed by the N-J (neighbor-joining) method where the number on the branch represented the bootstrap values. Subfamilies are marked in different colors, with each subfamily marked outside the circle as I, II, III, and IV. The red star, blue triangle, and green circle represented potato, rice, and *Arabidopsis* MATE proteins, respectively.

A phylogenetic tree was constructed by MEGA-X (v10.0.2) for the 64 StMATE proteins, 45 OsMATE proteins, and 56 AtDTX proteins that showed up after performing multiple sequence alignments, their protein sequence data are shown as Supplementary Table 3. According to the topology of the N-J phylogenetic tree as shown previously (Wang et al., 2016), the MATE genes of potato, rice, and *Arabidopsis* could be divided into four major subfamilies, namely, I, II, III, and IV, containing 23, 27, 4, and 10 StMATE genes, respectively (Figure 4).

### Transmembrane Helices, Exon–Intron Structure, and Motif Analysis of MATE Genes in Potato

**FIGURE 5:**
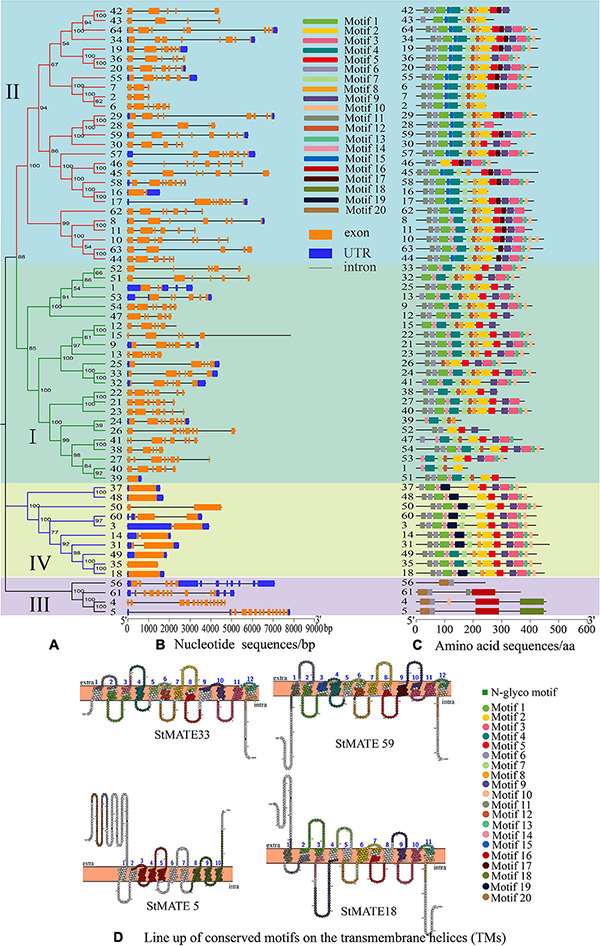
Analysis of motifs, transmembrane helices (TMs), and exon–intron structure of StMATE genes. The numbers 1–64 represent *StMATE1*–*StMATE64*. **(A)** Phylogenetic tree, the various subfamilies were numbered I, II, III, and IV. **(B)** Gene structures of StMATE genes, orange boxes, blue boxes, and lines represented exon, untranslated region (UTR), and introns, respectively. The lengths of the boxes and lines were scaled according to the gene length. **(C)** All motifs were identified by MEME (v4.12.0) according to the complete amino acid sequences. **(D)** Lineup of conserved motifs on the TMs. The transmembrane structure is marked with blue numbers, the conserved motif is indicated in different colors, and its related information is on the right.

The position information for the exons, intron, and untranslated region (UTR) on potato chromosomes was obtained through TBtools (v1.0692), which also helps construct the exon–intron structures and motif analysis diagram. The results of the gene structure analysis showed that genes in the same subfamily often had similar structures, but their intron lengths varied. Most members in subfamily I (21/23: 91.30%) had 5–7 exons, and the number of introns was similar to that of exon (Figure 5B), whereas 70.37% (19/27) MATE genes in subfamily II had 6–8 exons. The genes of subfamily III contained the greatest number of exons (10–13) among all. However, all the genes in subfamily IV either lacked introns or had less than three introns, *StMATE18*/*35*/*37*/*48*/*49* had no introns at all, *StMATE60* had three introns, and all other members had only one. Moreover, the numbers of motifs in StMATE genes in the same subfamily were highly similar (Figure 5A), with motifs in subfamily II being 8–15 in number. The minimum number of motifs for both I and IV was five, whereas the maximum number in I was 14 and in IV was 12. Subfamily III, however, contained only 2–5 motifs. The relevant information of 20 motifs is listed in Supplementary Table 2. Notably, we also analyzed the distribution of conserved motifs on the transmembrane helices (Figure 5D). *StMATE33*, *StMATE59*, *StMATE5*, and *StMATE18* contain all motifs of their respective subfamily.

### Promoter *Cis*-Acting Analysis

**FIGURE 6:**
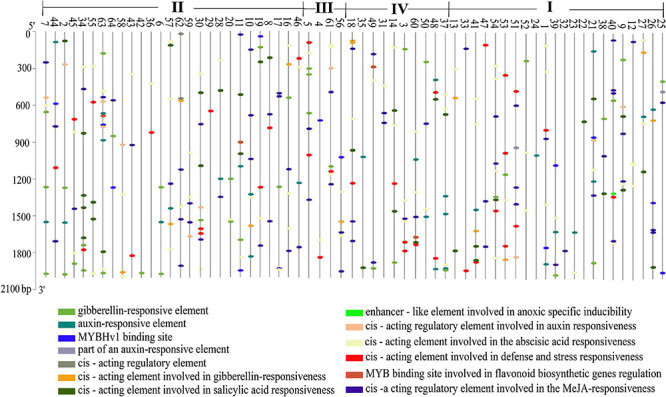
Analysis of *cis*-acting elements of StMATE genes. *Cis*-acting elements that existed in the 2,000-bp upstream region of StMATE genes were analyzed. Numbers 1–64 represent genes *StMATE1*–*StMATE64*, respectively; I, II, III, and IV represent the four subfamilies. Boxes with different colors at the bottom represent the *cis*-acting elements.

*Cis*-acting elements are the sites for specific binding of transcription factors and thus play an important role in regulating genes responsible for the growth, differentiation, and development of organisms, including plants. We extracted a 2,000-bp sequence in the upstream region of every StMATE gene and used PlantCARE (see text footnote 11) to identify all the *cis*-acting elements in these StMATE genes. We obtained 13 *cis*-acting elements according to their functional annotations and found that 61.54% (8/13) of them were related to hormone response (Figure 6). Interestingly, only *StMATE11*, *StMATE63* (II), and *StMATE60* (IV) contained a *cis*-acting, MYB binding site involved in flavonoid biosynthesis gene regulation.

### Expression Level Analysis of MATE Genes in Potato in Heavy Metal Stress

**FIGURE 7:**
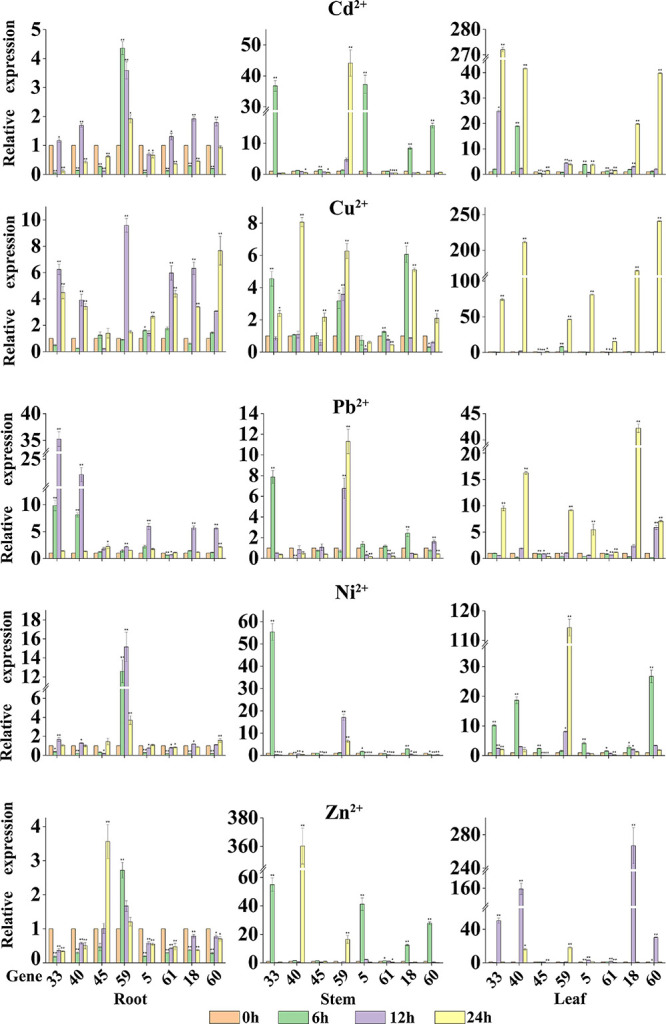
The relative expression levels of eight StMATE genes in response to the five heavy metals. The ordinate is the gene expression level; abscissa is the gene number. The height of the bar graphs represents the relative expression levels of the genes where the value of the expression is equal to the means (±SD) of three biological replicates. The gene names are shown on the *x* axis (*P < 0.05; **P < 0.01).

To further explore whether the expression levels of StMATE genes are affected by heavy metal stress, we selected a total of eight StMATE genes (two genes from each subfamily) after analyzing the phylogenetic tree and promoters and determined their relative expression levels by qRT-PCR in roots, stems, and leaves after Cu^2+^, Cd^2+^, Zn^2+^, Ni^2+^, and Pb^2+^ treatment for 0, 6, 12, and 24 h. The expression level at 0 h of treatment was taken as 1 (Figure 7). The results of qRT-PCR analysis showed that, compared with control (0 h), in the same tissue, the time for StMATE genes to reach a higher expression level was similar, i.e., the time for most genes to reach the highest expression level in root, stem, and leaf tissues was 12, 6, and 24 h, respectively, under these five heavy metal stress conditions. In leaf tissues, however, in the case of Ni^2+^ stress, the time for the eight StMATE genes to reach the highest expression level was 6 h and for Zn^2+^ stress, it was 12 h. In leaf tissue, the expression levels of *StMATE33* and *StMATE40* (I) under Cd^2+^ stress were upregulated to 272.1 and 41.6 times, respectively, than that in the control, after a 24-h treatment (*P* > 0.01). The levels for them in stem tissues were increased to 36.8 and 1.2 times that of the control (6 h treatment), and the expression levels in the root tissues were 1.2 and 1.7 times that of the control for a 12-h treatment (not significant). Under Cu^2+^, Pb^2+^, and Ni^2+^ stress, the relative expression levels of *StMATE33* and *StMATE40* in each tissue were similar to that of Cd^2+^ treatment. For Zn^2+^ stress of 12 h, the expression levels of *StMATE33* and *StMATE40* in leaf tissues were 50.4 and 159.2 times, respectively, that of the control; in stem tissue, a Zn^2+^ treatment for 24 h increased the expression level of *StMATE40* gene highly significantly to 360.3. Under Ni^2+^ stress conditions, the expression levels of *StMATE45* and *StMATE59* (II) were quite different, where *StMATE45* showed lower expression levels in roots, stems, and leaves. On the contrary, the highest relative expression levels of *StMATE59* in roots, stems, and leaves were 15.2, 17, and 114, respectively, which were extremely significantly upregulated. The results indicated that the expression levels of genes in the same subfamily could be significantly different even under the same stress conditions. In addition, the expression levels of *StMATE45* and *StMATE59* under different stresses in various tissues were similar to Ni^2+^ stress. *StMATE5* and *StMATE61* (III) showed a maximum response to Cu^2+^ stress compared with the other heavy metal stresses. Under Cu^2+^ stress treatment for 24 h, the relative expression levels of *StMATE5* and *StMATE61* in leaf tissue were up by 81.2 and 15.4 times, respectively, than that of the control. In the stem tissue, they were up only by 0.7 and 1.3 times that of the control with a 6-h treatment; in root tissue, however, after 12 h of Cu^2+^ stress treatment, the expression levels *StMATE5* and *StMATE61* were 1.4 and 6 times that of the control. Moreover, the expression levels of *StMATE5* and *StMATE61*, under the other four heavy metal stress treatments, had a similar trend to Cu^2+^ stress. Finally, for *StMATE18* and *StMATE60* (IV) under a Zn^2+^ stress of 12 h, the gene expression levels in leaf tissue were 266.8 and 30.33 times higher, respectively. In the stem tissue, however, in a Zn^2+^ stress for 6 h, the gene expression levels increased 12.49 and 27.78 times, whereas in root tissues, under a Zn^2+^ stress of 12 h, the gene expression levels were found to be lower than the control. In the stem and leaf tissues, *StMATE18* and *StMATE60* had the highest expression levels after stress treatment for 6 h, while it was 24 h for the root tissue. For the other three heavy metal stress treatments, the time for *StMATE18* and *StMATE60* to reach higher expression levels in root, stem, and leaf tissues was 12, 6, and 24 h, respectively.

All results of our study indicated that certain genes have a higher expression level in specific tissues and under specific stress conditions. For example, the expression levels of most of the eight StMATE genes in each tissue followed a trend of leaf > stem > root. However, under Pb^2+^ stress, the expression levels of *StMATE33* and *StMATE40* (I) showed a trend of root > leaf > stem. Additionally, the overall expression levels of *StMATE33* and *StMATE40* under Zn^2+^ treatment were stem > leaf > root. The results show that the response degree of the same gene to different treatments is different, and the expression levels are related to the organ type and the time of stress treatment.

## Discussion

Multidrug and toxic compound extrusion transporters are widely present in plant cells, mainly membranes, and play an important role in the efflux of plant secondary metabolites and toxic compounds (Lu et al., 2018). In our present study, a total of 64 members of the potato MATE family were identified (Supplementary Table 1), and the results of their subcellular localization prediction showed that 98.44% of them too were in the plasma membrane. It reinforces that fact that MATE proteins are indeed membrane transporters, acting as gatekeepers for cells by regulating the inflow of useful substances and the exudation of the harmful ones (Upadhyay et al., 2019). Furthermore, they are distributed on 12 chromosomes in *Cloud S. tuberosum* (Figure 1) and comprise four major subfamilies (I–IV) based on their evolutionary relationships (Figure 4). These observations are consistent with the previous classification of *MATE* genes in rice and *Arabidopsis* (Wang et al., 2016).

Syntenic analysis showed that there are seven pairs of collinearity genes, and they predominantly amplify through gene duplication (Figure 2). Among them, five pairs of collinearity genes belong to subfamily I and two pairs belong to subfamily II. Our results also indicate that the fragment duplication also contributes to the evolution of genes in subfamilies I and II. The result showed that some StMATE genes can cause the amplification on different chromosomes through gene duplication. As protein functions are affected by their structures (Qin et al., 2019), we analyzed the gene structure and motifs for these genes and found that StMATE genes in the same subfamily had similar exon–intron structure and shared conserved motifs (Figure 5) as well. The loss and gain of introns might reflect the evolutionary trend of gene families (Rogozin et al., 2003), which can indicate that genes in the same evolutionary branch not only have similar gene structures but also have similar functions, such as most genes in subfamily IV completely lacked or had only one intron, indicating that their amplification might be happening differently from genes in other subfamilies.

Additionally, the result of motif analysis among the four (I–IV) subfamilies showed that all StMATE genes contain motif6, and the composition and number of conserved motifs in subfamilies I, II, and IV are quite similar (between 5 and 15). It is worth noting that each subfamily contains unique conservative motifs. For example, motif17 and motif19 exist in subfamily II and subfamily IV, respectively, while motif16 and motif20 are unique to subfamily III. Motif17 lines up with TM9 of *StMATE59*. Some amino acid residues of motif19 are arranged on TM4 of *StMATE18*, but motif20 is arranged on the outside of the membrane of *StMATE5*, and motif16 penetrates TM2–TM5 of *StMATE5* (Figure 5D). The presence of conserved amino acid motifs helps in compartmentalization of proteins into subfamilies and may have significance to the function of the proteins within the family (Seo and Kim, 2018). Similar results were found for DTX/MATE protein in flax (Ali et al., 2020). As phytohormones are very crucial for MATE proteins to extrude heavy metal and transmit certain disease resistance signals (Serrano et al., 2013; Garcia-Oliveira et al., 2018; Tegli et al., 2020). We selected 13 cis-acting elements, based on their functional annotations, and found that 61.54% (8/13) of them were also related to hormone response (Figure 6). Interestingly, *StMATE11* and *StMATE63* (from subfamily II) contain *cis* elements for the MYB binding site, near genes involved in the flavonoid biosynthesis regulation. The results widely suggest that the two genes may be related to the transport of flavonoids.

As phylogenetic analysis of the genes can help to conjecture their function in a specific species (Pandey et al., 2016), we performed the same analysis (Figure 4) on the 64 StMATE genes and discovered that they comprise four major subfamilies (I–IV). Some members of the subfamily I clustered together with *AtDTX1* and may be involved in the synthesis of alkaloids, auxins, and the transportation of toxic compounds such as cadmium (Li et al., 2002). Other members of the lot gathered together with *ALF5* (*AtDTX19*) and *AtDTX18*. The former transports tetramethylammonium (Diener and Fink, 2001), and the latter can enhance a plant’s resistance toward a disease (Dobritzsch et al., 2016). Members of subfamily II seem to have similar functions as *TT12*, i.e., to participate in the transport of flavonoids (Marinova et al., 2007), whereas genes in subfamily III gathered with genes such as *FRD3* (*AtDTX43*) and *EDS5* (*AtDTX47*), which are involved in transport of iron (Durrett et al., 2007; Pineau et al., 2012) and salicylic acid (Nawrath et al., 2002), respectively. Interestingly, the kinship of genes in subfamily III is relatively a closer one (bootstrap values >80). So, it can be speculated that proteins in this subfamily may function like FRD3 and EDS5. Ultimately, members of subfamily IV appeared in the same branch as AtDTX50 and thus may be involved in the efflux of ABA (Zhang et al., 2014).

**FIGURE 8:**
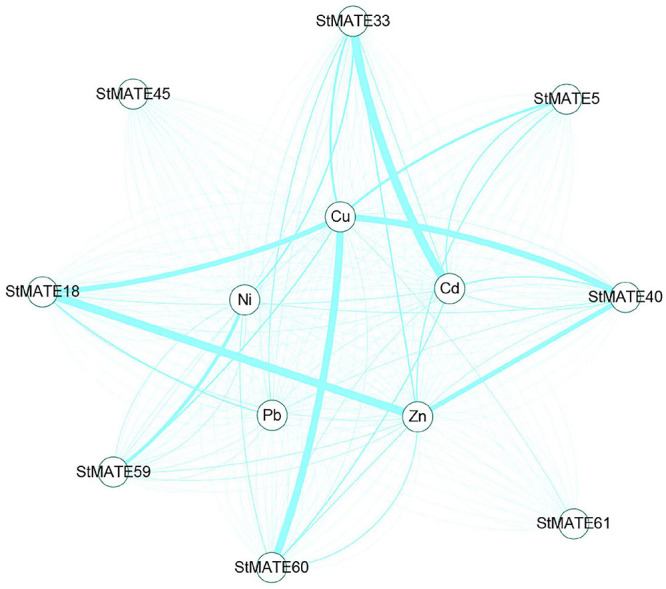
Analysis of co-expression network of StMATE genes. The thicker the line between metals and genes indicates the higher expression of the gene under the metal stress condition.

The results of qRT-PCR analysis indicate that the eight selected StMATE genes were expressed in root, stem, and leaf tissues under five heavy metal stress conditions. Quite notably, in the same tissue, genes of the same subfamily have similar expression levels if undergoing the same stress conditions. It is consistent with the results of gene structure and motif analysis (Figure 5), which also indicates a similar structure and function for genes in the same subfamily. In addition, under all stress conditions, the overall expression level of genes in each tissue follows a trend of leaf > stem > root. However, in different plant tissues, genes from different subfamilies respond to stresses with variant degrees. For example, the expression levels of *StMATE33* under Cd^2+^ stress for 24 h were increased to the highest amount 272.1 compared to unstressed controls (0 h); under Cu^2+^ and Zn^2+^ stress conditions, *StMATE40* is extremely significantly upregulated. Moreover, *StMATE33* and *StMATE40* are in the same subfamily as *AtDTX1* is in the phylogenetic tree (subfamily I). In *Arabidopsis*, *AtDTX1* can participate in the transportation of toxic compounds such as alkaloids, phytohormones, and cadmium (Kuroda and Tsuchiya, 2009). Therefore, we speculate that *StMATE33* may be playing a crucial role in the responses toward Cd^2+^ stress in *Cloud S*. *tuberosum*. Quite consistently, the expression level of *StMATE40* was also significantly induced by Cu^2+^ and Zn^2+^. This result could be attributed to the fact that the three metals have similar structures, while zinc (atomic number 30) and copper (atomic number 29) are in adjacent periods as well in the periodic table (Supanchaiyamat and Hunt, 2019). Compared with the control, Ni^2+^ stress significantly increased the expression levels of *StMATE59* (subfamily II), while *StMATE18* and *StMATE60* (subfamily IV) were significantly upregulated under Cu^2+^ stress. Most importantly, we can get more useful information from Figure 8. Under Cu^2+^ stress, the expression levels of *StMATE18*/*60*/*40*/*33*/*5* were all significantly upregulated, and the expression levels of *StMATE18*/*40* were significantly upregulated under Zn^2+^ stress. Differently, only the expression of *StMATE33* was extremely significantly upregulated under Cd^2+^ stress. The results indicate that there may be a synergistic effect between *StMATE18*/*60*/*40*/*33*/*5* under Cu^2+^ stress conditions, and *StMATE33* is very important for cadmium detoxification. Copper and zinc are important components of superoxide dismutase (SOD) and ceruloplasmin, which are important in many metabolic processes and enzyme systems (Airede, 1993). Based on this, we speculate that *StMATE18*/*60*/*40*/*33*/*5* are very important for the growth of plants.

Chen et al. (2015) found that *VcMATE1* and *VcMATE4* are in the same evolutionary branch as *AtDTX1* and speculated that they play an important role in the transportation of alkaloids and the efflux of xenobiotics or toxic cations. *LuDTX71* and *LuDTX73* genes can enhance plant tolerance to Cd, cold, and salt stress (Ali et al., 2020). These results, combined with our research results, strongly support that the StMATE genes may be involved in the detoxification of cadmium ions. In particular, the expression level of *CaMATE01/28* is upregulated under hormone stress conditions, and phylogenic analysis indicated that they are in the same evolutionary branch as *AtDTX1* (Chen Q. et al., 2020). It indicates that hormone *cis*-acting elements are essential for the MATE gene family to respond to adversity stress. And MATE transporters can improve the ability to adapt to adversity by promoting the efflux of phytohormone (Zhang et al., 2014; Yokosho et al., 2016; Wang et al., 2017).

On the whole, we have identified 64 StMATE genes from the potato genome, divided them into four major subfamilies, and analyzed the expression levels of eight StMATE genes in heavy metal conditions. Analysis of qRT-PCR revealed the significant changes in expression levels of eight StMATE genes under five heavy metal stresses. In particular, Cu^2+^ stress quite significantly upregulated the expression levels of most genes. However, the levels of *StMATE33* (I) were quite abundantly induced by Cd^2+^, and *StMATE18*/*60*/*40*/*33*/*5* were significantly upregulated under Cu^2+^ stress, while *StMATE59* (II) was significantly induced by Ni^2+^ stress. Our detailed findings lay down the initial paths to help researchers in building future projects that would uncover the molecular mechanisms of StMATE genes, in response to heavy metals stress, in the commercially important food crop *Cloud S. tuberosum*.

## Data Availability Statement

The original contributions presented in the study are included in the article/Supplementary Material, further inquiries can be directed to the corresponding author/s.

## Author Contributions

TH and YH conceived the idea of the experiments. YH, DL, LM, and WT carried out the experiments. YH, DL, GH, and WT analyzed the data. YH wrote the manuscript. All authors read and approved the final manuscript.

## Conflict of Interest

The authors declare that the research was conducted in the absence of any commercial or financial relationships that could be construed as a potential conflict of interest.

## Footnotes

1 http://plants.ensembl.org/index.html

2 http://pfam.xfam.org/family/PF01554

3 https://www.omicsclass.com/article/681

4 https://www.ncbi.nlm.nih.gov/cdd/

5 http://pfam.xfam.org/

6 https://web.expasy.org/protparam/

7 http://www.csbio.sjtu.edu.cn/bioinf/plant-multi/

8 http://mg2c.iask.in/mg2c_v2.0/

9 https://www.omicsclass.com/article/671

10 http://wlab.ethz.ch/protter/start/

11 http://bioinformatics.psb.ugent.be/webtools/plantcare/html/

## References

[B1] Airede A. K. (1993). Copper, zinc and superoxide dismutase activities in premature infants: a review. *East Afr. Med. J.* 70 441–444. 8293704

[B2] Ali E. Saand M. A. Khan A. R. Shah J. M. Feng S. Ming C. (2020). Genome-wide identification and expression analysis of detoxification efflux carriers (DTX) genes family under abiotic stresses in flax. *Physiol. Plant* 171 483–501. 10.1111/ppl.13105 32270877

[B3] Chen C. Chen H. Zhang Y. Thomas H. R. Frank M. H. He Y. (2020). TBtools: an integrative toolkit developed for interactive analyses of big biological data. *Mol. Plant* 13 1194–1202. 10.1016/j.molp.2020.06.009 32585190

[B4] Chen L. Liu Y. Liu H. Kang L. Geng J. Gai Y. (2015). Identification and expression analysis of MATE genes involved in flavonoid transport in blueberry plants. *PLoS One* 10:e118578. 10.1371/journal.pone.0118578 25781331 PMC4363705

[B5] Chen Q. Wang L. Liu D. Ma S. Dai Y. Zhang Z. (2020). Identification and expression of the multidrug and toxic compound extrusion (MATE) gene family in *Capsicum annuum* and *Solanum tuberosum*. *Plants* 9:1448. 10.3390/plants9111448 33120967 PMC7716203

[B6] Das N. Bhattacharya S. Bhattacharyya S. Maiti M. K. (2018). Expression of rice MATE family transporter OsMATE2 modulates arsenic accumulation in tobacco and rice. *Plant. Mol. Biol.* 98 101–120. 10.1007/s11103-018-0766-1 30121733

[B7] Diener A. C. Fink G. G. R. (2001). Arabidopsis ALF5, a multidrug efflux transporter gene family member, confers resistance to toxins. *Plant Cell* 13 1625–1637. 11449055 10.1105/TPC.010035 PMC139545

[B8] Dobritzsch M. Lubken T. Eschen-Lippold L. Gorzolka K. Blum E. Matern A. (2016). MATE transporter-dependent export of hydroxycinnamic acid amides. *Plant Cell* 28 583–596. 10.1105/tpc.15.00706 26744218 PMC4790871

[B9] Durrett T. P. Gassmann W. Rogers E. E. (2007). The FRD3-mediated efflux of citrate into the root vasculature is necessary for efficient iron translocation. *Plant Physiol.* 144 197–205. 10.1104/pp.107.097162 17351051 PMC1913786

[B10] Garcia-Oliveira A. L. Benito C. Guedes-Pinto H. Martins-Lopes P. (2018). Molecular cloning of TaMATE2 homoeologues potentially related to aluminium tolerance in bread wheat (*Triticum aestivum* L.). *Plant Biol.* 20 817–824. 10.1111/plb.12864 29908003

[B11] Gomez C. Terrier N. Torregrosa L. Vialet S. Fournier-Level A. Verriès C. (2009). Grapevine MATE-type proteins act as vacuolar H+-dependent acylated anthocyanin transporters. *Plant Physiol.* 150 402–415. 10.1104/pp.109.135624 19297587 PMC2675721

[B12] He G. Qin L. Tian W. Meng L. He T. Zhao D. (2020). Heavy metal Transporters-Associated proteins in *Solanum tuberosum*: genome-wide identification, comprehensive gene feature, evolution and expression analysis. *Genes* 11:1269. 10.3390/genes11111269 33126505 PMC7694169

[B13] He G. Tian W. Qin L. Meng L. Wu D. Huang Y. (2021). Identification of novel heavy metal detoxification proteins in *Solanum tuberosum*: insights to improve food security protection from metal ion stress. *Sci. Total Environ.* 779:146197. 10.1016/j.scitotenv.2021.146197 33744586

[B14] Kryvoruchko I. S. Routray P. Sinharoy S. Torresjerez I. Finney L. A. Nakashima J. (2018). An iron-activated citrate transporter, MtMATE67, is required for symbiotic nitrogen fixation. *Plant Physiol.* 176:1538. 10.1104/pp.17.01538 PMC5841734 29284744

[B15] Kuroda T. Tsuchiya T. (2009). Multidrug efflux transporters in the MATE family. *Biochim. Biophys. Acta* 1794 763–768. 10.1016/j.bbapap.2008.11.012 19100867

[B16] Li L. He Z. Pandey G. K. Tsuchiya T. Luan S. (2002). Functional cloning and characterization of a plant efflux carrier for multidrug and heavy metal detoxification. *J. Biol. Chem.* 277 5360–5368. 10.1074/jbc.M108777200 11739388

[B17] Li Y. He H. He L. F. (2019). Genome-wide analysis of the MATE gene family in potato. *Mol. Biol. Rep.* 46 403–414. 10.1007/s11033-018-4487-y 30446960

[B18] Liu J. Li Y. Wang W. Gai J. Li Y. (2016). Genome-wide analysis of MATE transporters and expression patterns of a subgroup of MATE genes in response to aluminum toxicity in soybean. *BMC Genom.* 17:223. 10.1186/s12864-016-2559-8 26968518 PMC4788864

[B19] Livak K. J. Schmittgen T. D. (2001). Analysis of relative gene expression data using real-time quantitative PCR and the 2^−ΔΔ*C*_T_^ Method. *Methods* 25 402–408. 10.1006/meth.2001.1262 11846609

[B20] Lu M. (2016). Structures of multidrug and toxic compound extrusion transporters and their mechanistic implications. *Channels* 10 88–100. 10.1080/19336950.2015.1106654 26488689 PMC4960993

[B21] Lu P. Magwanga R. O. Guo X. Kirungu J. N. Lu H. Cai X. (2018). Genome-Wide analysis of multidrug and toxic compound extrusion (MATE) family in *Gossypium raimondii* and *Gossypium arboreum* and its expression analysis under salt, cadmium, and drought stress. *G3* 8 2483–2500. 10.1534/g3.118.200232 29794162 PMC6027885

[B22] Lu P. Magwanga R. O. Kirungu J. N. Hu Y. Dong Q. Li Y. (2019). Overexpression of cotton a DTX/MATE gene enhances drought, salt, and cold stress tolerance in transgenic *Arabidopsis*. *Front. Plant Sci.* 10:299. 10.3389/fpls.2019.00299 30930923 PMC6423412

[B23] Marinova K. Pourcel L. Weder B. Schwarz M. Barron D. Yang M. (2007). The *Arabidopsis* MATE transporter TT12 acts as a vacuolar flavonoid/H+ -antiporter active in proanthocyanidin-accumulating cells of the seed coat. *Plant Cell* 19 2023–2038. 10.1105/tpc.106.046029 17601828 PMC1955721

[B24] Maron L. G. Pineros M. A. Guimaraes C. T. Magalhaes J. V. Pleiman J. K. Mao C. (2010). Two functionally distinct members of the MATE (multi-drug and toxic compound extrusion) family of transporters potentially underlie two major aluminum tolerance QTLs in maize. *Plant J.* 61 728–740. 10.1111/j.1365-313X.2009.04103.x 20003133

[B25] Min X. Jin X. Liu W. Wei X. Zhang Z. Ndayambaza B. (2019). Transcriptome-wide characterization and functional analysis of MATE transporters in response to aluminum toxicity in *Medicago sativa* L. *Peer J.* 7:e6302. 10.7717/peerj.6302 PMC6360082 30723620

[B26] Morita Y. Kodama K. Shiota S. Mine T. Kataoka A. Mizushima T. (1998). NorM, a putative multidrug efflux protein, of *Vibrio parahaemolyticus* and its homolog in *Escherichia coli*. *Antimicrob. Agents Chemother.* 42 1778–1782. 10.1128/AAC.42.7.1778 9661020 PMC105682

[B27] Mustafa G. Komatsu S. (2016). Toxicity of heavy metals and metal-containing nanoparticles on plants. *Biochim. Biophys. Acta* 1864 932–944. 10.1016/j.bbapap.2016.02.020 26940747

[B28] Nawrath C. Heck S. Parinthawong N. Métraux J. P. (2002). EDS5, an essential component of salicylic acid-dependent signaling for disease resistance in *Arabidopsis*, is a member of the MATE transporter family. *Plant Cell* 14 275–286. 11826312 10.1105/tpc.010376 PMC150564

[B29] Omote H. Hiasa M. Matsumoto T. Otsuka M. Moriyama Y. (2006). The MATE proteins as fundamental transporters of metabolic and xenobiotic organic cations. *Trends Pharmacol. Sci.* 27 587–593. 10.1016/j.tips.2006.09.001 16996621

[B30] Pandey A. Misra P. Alok A. Kaur N. Sharma S. Lakhwani D. (2016). Genome-wide identification and expression analysis of homeodomain leucine zipper subfamily IV (HDZ IV) gene family from *Musa accuminata*. *Front. Plant Sci.* 7:20. 10.3389/fpls.2016.00020 26870050 PMC4740955

[B31] Pineau C. Loubet S. Lefoulon C. Chalies C. Fizames C. Lacombe B. (2012). Natural variation at the FRD3 MATE transporter locus reveals cross-talk between Fe homeostasis and Zn tolerance in *Arabidopsis thaliana*. *PLoS Genet.* 8:e1003120. 10.1371/journal.pgen.1003120 23236296 PMC3516540

[B32] Qin M. Luo W. Zheng Y. Guan H. Xie X. (2019). Genome-wide identification and expression analysis of the PHD-finger gene family in *Solanum tuberosum*. *PLoS One* 14:e226964. 10.1371/journal.pone.0226964 31881057 PMC6934267

[B33] Rascio N. Navari-Izzo F. (2011). Heavy metal hyperaccumulating plants: how and why do they do it? And what makes them so interesting? *Plant Sci.* 180 169–181. 10.1016/j.plantsci.2010.08.016 21421358

[B34] Rogozin I. B. Wolf Y. I. Sorokin A. V. Mirkin B. G. Koonin E. V. (2003). Remarkable interkingdom conservation of intron positions and massive, lineage-specific intron loss and gain in eukaryotic evolution. *Curr. Biol.* 13 1512–1517. 10.1016/s0960-9822(03)00558-x 12956953

[B35] Santos A. Chaves-Silva S. Yang L. Maia L. Chalfun-Junior A. Sinharoy S. (2017). Global analysis of the MATE gene family of metabolite transporters in tomato. *BMC Plant Biol.* 17:185. 10.1186/s12870-017-1115-2 29084510 PMC5663081

[B36] Seo M. H. Kim P. M. (2018). The present and the future of motif-mediated protein-protein interactions. *Curr. Opin. Struct. Biol.* 50 162–170. 10.1016/j.sbi.2018.04.005 29730529

[B37] Serrano M. Wang B. Aryal B. Garcion C. Abou-Mansour E. Heck S. (2013). Export of salicylic acid from the chloroplast requires the multidrug and toxin extrusion-like transporter EDS5. *Plant Physiol.* 162 1815–1821. 10.1104/pp.113.218156 23757404 PMC3729763

[B38] Shannon P. Markiel A. Ozier O. Baliga N. S. Wang J. T. Ramage D. (2003). Cytoscape: a software environment for integrated models of biomolecular interaction networks. *Genome Res.* 13 2498–2504. 10.1101/gr.1239303 14597658 PMC403769

[B39] Shoji T. Inai K. Yazaki Y. Sato Y. Takase H. Goto Y. (2009). Multidrug and toxic compound extrusion-type transporters implicated in vacuolar sequestration of nicotine in tobacco roots. *Plant Physiol.* 149 708–718. 10.1104/pp.108.132811 19098091 PMC2633862

[B40] Sivaguru M. Liu J. Kochian L. V. (2013). Targeted expression of SbMATE in the root distal transition zone is responsible for sorghum aluminum resistance. *Plant J.* 76 297–307. 10.1111/tpj.12290 23865685

[B41] Sun W. Ma Z. Chen H. Liu M. (2019). MYB gene family in potato (*Solanum tuberosum* l.): genome-wide identification of hormone-responsive reveals their potential functions in growth and development. *Intern. J. Mol. Sci.* 20:4847. 10.3390/ijms20194847 31569557 PMC6801432

[B42] Sun X. Gilroy E. M. Chini A. Nurmberg P. L. Hein I. Lacomme H. (2011). ADS1 encodes a MATE-transporter that negatively regulates plant disease resistance. *New Phytol.* 192 471–482. 21762165 10.1111/j.1469-8137.2011.03820.x

[B43] Supanchaiyamat N. Hunt A. J. (2019). Conservation of critical elements of the periodic table. *Chemsuschem* 12 397–403. 10.1002/cssc.201802556 30604524

[B44] Tegli S. Bini L. Calamai S. Cerboneschi M. Biancalani C. (2020). A MATE transporter is involved in pathogenicity and IAA homeostasis in the hyperplastic plant pathogen *Pseudomonas savastanoi* pv. Nerii. *Microorganisms* 8:156. 10.3390/microorganisms8020156 31979049 PMC7074806

[B45] Tian W. He G. Qin L. Li D. Meng L. Huang Y. (2021). Genome-wide analysis of the NRAMP gene family in potato (*Solanum tuberosum*): identification, expression analysis and response to five heavy metals stress. *Ecotoxicol. Environ. Saf.* 208:111661. 10.1016/j.ecoenv.2020.111661 33396171

[B46] Tiwari M. Sharma D. Singh M. Tripathi R. D. Trivedi P. K. (2014). Expression of OsMATE1 and OsMATE2 alters development, stress responses and pathogen susceptibility in *Arabidopsis*. *Sci. Rep.* 4:3964. 10.1038/srep03964 PMC3912489 24492654

[B47] Upadhyay N. Kar D. Deepak M. B. Nanda S. Rahiman R. Panchakshari N. (2019). The multitasking abilities of MATE transporters in plants. *J. Exp. Bot.* 70 4643–4656. 10.1093/jxb/erz246 31106838

[B48] Wang J. Hou Q. Li P. Yang L. Sun X. Bhagavatula L. (2017). Diverse functions of multidrug and toxin extrusion (MATE) transporters in citric acid efflux and metal homeostasis in *Medicago truncatula*. *Plant J.* 90 79–95. 10.1111/tpj.13471 28052433

[B49] Wang L. Bei X. Gao J. Li Y. Yan Y. Hu Y. (2016). The similar and different evolutionary trends of MATE family occurred between rice and *Arabidopsis thaliana*. *BMC Plant Biol.* 16:207. 10.1186/s12870-016-0895-0 27669820 PMC5037600

[B50] Wang S. Zhang N. Zhu X. Yang J. Li S. Che Y. (2019). Identification and expression analysis of StGRAS gene family in potato (*Solanum tuberosum* L.). *Comput. Biol. Chem.* 80 195–205. 10.1016/j.compbiolchem.2019.03.020 30978571

[B51] Wang Y. Tang H. Debarry J. D. Tan X. Li J. Wang X. (2012). MCScanX: a toolkit for detection and evolutionary analysis of gene synteny and collinearity. *Nucleic Acids Res.* 40:e49. 10.1093/nar/gkr1293 22217600 PMC3326336

[B52] Xu L. Shen Z. L. Chen W. Si G. Y. Meng Y. Guo N. (2019). Phylogenetic analysis of upland cotton MATE gene family reveals a conserved subfamily involved in transport of proanthocyanidins. *Mol. Biol. Rep.* 46 161–175. 10.1007/s11033-018-4457-4 30467666

[B53] Yokosho K. Yamaji N. Fujii-Kashino M. Ma J. F. (2016). Retrotransposon-Mediated aluminum tolerance through enhanced expression of the citrate transporter OsFRDL4. *Plant Physiol.* 172 2327–2336. 10.1104/pp.16.01214 27744299 PMC5129714

[B54] Yokosho K. Yamaji N. Ma J. F. (2011). An Al-inducible MATE gene is involved in external detoxification of Al in rice. *Plant J.* 68 1061–1069. 10.1111/j.1365-313X.2011.04757.x 21880027

[B55] Yokosho K. Yamaji N. Ueno D. Mitani N. Ma J. F. (2009). OsFRDL1 is a citrate transporter required for efficient translocation of iron in rice. *Plant Physiol.* 149 297–305. 10.1104/pp.108.128132 19011004 PMC2613705

[B56] Zhang H. Zhu H. Pan Y. Yu Y. Luan S. Li L. (2014). A DTX/MATE-type transporter facilitates abscisic acid efflux and modulates ABA sensitivity and drought tolerance in *Arabidopsis*. *Mol. Plant* 7 1522–1532. 10.1093/mp/ssu063 24851876

[B57] Zhao P. Wang D. Wang R. Kong N. Zhang C. Yang C. (2018). Genome-wide analysis of the potato Hsp20 gene family: identification, genomic organization and expression profiles in response to heat stress. *BMC Genom.* 19:61. 10.1186/s12864-018-4443-1 29347912 PMC5774091

[B58] Zhu H. Wu J. Jiang Y. Jin J. Zhou W. Wang Y. (2016). Genomewide analysis of MATE-type gene family in maize reveals microsynteny and their expression patterns under aluminum treatment. *J. Genet.* 95 691–704. 10.1007/s12041-016-0686-2 27659341

